# The Histopathological Spectrum of Scrotal Lesions in a Tertiary Care Hospital: A Cross-Sectional Study

**DOI:** 10.7759/cureus.52767

**Published:** 2024-01-22

**Authors:** Apara Desai, Anne Wilkinson

**Affiliations:** 1 Pathology, NKP Salve Institute of Medical Sciences & Research Centre and Lata Mangeshkar Hospital, Nagpur, IND

**Keywords:** testicular tumours, para-testicular lesions, seminoma, scrotal lesions, histopathological spectrum

## Abstract

Background: The incidence and clinical presentation of testicular and paratesticular lesions are variable. A preoperative diagnosis is often difficult with only a clinical examination. The diagnosis of testicular lesions is mainly based on histological investigation, despite advances in imaging and tumor marker testing. This study aimed to document the histopathological spectrum of scrotal lesions, including testicular and paratesticular lesions.

Aim: The study aimed to research the histopathological spectrum of scrotal lesions.

Settings and design: This was a cross-sectional study conducted at NKP Salve Institute of Medical Sciences & Research Centre and Lata Mangeshkar Hospital, a tertiary care hospital in Nagpur, India.

Materials and methods: Following the institutional ethics committee's approval, a two-year cross-sectional study was carried out in the tertiary care hospital. Seventy operated scrotal specimens sent for histopathological examination were included in the study. The clinical details and investigations of the patients, as well as the gross and histopathological findings of all the specimens, were studied carefully.

Statistical analysis: The clinical details and gross and histopathological findings were noted in a proforma, entered in a Microsoft Excel sheet (Microsoft Corp., Redmond, WA), and verified. The data were presented in a tabular form using tablets, pie charts, and bar diagrams. The collected data were analyzed and presented in percentages and frequencies.

Results: The present study evaluated the histopathological spectrum of scrotal lesions in 70 operated scrotal masses. The mean age of the participants in the study was 46.55 ± 18.69 years, with the youngest patient at four years and the oldest being 88 years of age. Sixty-six (80%) of the 70 cases were of non-neoplastic lesions, while 14 (20%) were of neoplastic lesions. Testicular atrophy (16 cases) was the most common non-neoplastic lesion. The most frequent neoplastic lesion in the present study was a seminoma (seven cases).

Conclusion: This study strongly recommends routine histopathological examination of all scrotal specimens for the detection of various testicular and paratesticular lesions, as well as neoplasms. Histopathology not only provides a tissue diagnosis in scrotal disorders, but it also adds to understanding etiopathogenesis and can aid in the development of future treatment options.

## Introduction

The testes, epididymis, and distal spermatic cord are located in the cutaneous fibromuscular sac known as the scrotum. Testes are paired, and they lie within the scrotum attached to the spermatic cord [1]. Testicular disease is a common health problem. Testicular disorders could be congenital or acquired lesions that are inflammatory or neoplastic [2]. Testicular tumors can be of two types: benign or malignant. Scrotal swelling, pain, and a palpable mass are the usual presenting features [3]. Inflammatory intrascrotal conditions can lead to sepsis, testicular loss, or decreased fertility, but are rarely life-threatening [4]. Neoplastic conditions from paratesticular structures like lipoma, leiomyoma, and hemangioma often imitate malignancy, which may prompt an unwarranted radical orchidectomy [5]. Testicular cancer represents 1% of malignancies in men [2]. Testicular and paratesticular malignancies are the most common solid malignancies in males aged between 15 and 34 years [2]. With only a clinical examination, preoperative diagnosis is frequently difficult [5]. As a result, a definitive diagnosis must rely on a histopathologic examination. Hence, we aimed to study the histopathological spectrum of scrotal lesions, including testicular and paratesticular lesions.

## Materials and methods

Study subjects and methods

This study was conducted at NKP Salve Institute of Medical Sciences & Research Centre and Lata Mangeshkar Hospital, a tertiary care hospital in Nagpur, India after approval from the institute's ethics committee (approval number: 106/2021). The study population included 70 patients who gave consent and underwent operative surgeries for scrotal masses. Inclusion criteria were 1. All scrotal specimens, which included testicular and paratesticular lesions received in the pathology department over two years and all patients who consented to being part of the study. The exclusion criteria were not applicable. All consecutive cases fitting the inclusion criteria were included in the study. All testicular and scrotal specimens were studied grossly as well as histologically. A detailed study of the specimens with respect to size, external surface, and presence of tumors with respect to their location, extension, consistency, and presence of hemorrhage and necrosis was done. All the findings were noted. Appropriate sections from the tumor or lesion as well as adjoining normal tissue were taken.

The clinical details and gross and histopathological findings were noted in a proforma. The data were entered in a Microsoft Excel sheet (Microsoft Corp., Redmond, WA), and then verified. The data were presented in a tabular form using tablets, pie charts, and bar diagrams. The collected data were analyzed in percentages and frequencies. Categorical variables were presented in numbers and percentages (%).

## Results

Out of the 70 cases involved in the study, the age group of 51-60 years had the most cases (23.8%). The mean age of the patients was 46.55 years. The youngest patient was four years old, while the oldest was 88. The most common clinical complaint was scrotal/inguinoscrotal swelling (80%), followed by pain (41.43%). Fever was associated with 12.86% of the cases, while cryptorchidism was the presenting complaint in 8.5% of cases (Table 1).

**Table 1:** Presenting complaints of the patients

	Frequency	Percentage
Pain	29	41.43%
Swelling (scrotal/inguinoscrotal)	56	80.00%
Fever	09	12.86%
Cryptorchidism	06	8.57%
Discharge from the scrotal area	01	1.42%
Open wound on the scrotum	03	4.26%

Open wounds on the scrotum and discharge from the scrotal area were other complaints. Prophylactic orchiectomy was performed in one known case of prostate adenocarcinoma. Right-sided lesions (64.28%) were more common than left-sided lesions (28.57%) in this study. Bilateral involvement was seen in five cases (7.14%).

Hydrocele was the most prevalent non-neoplastic lesion identified radiologically (18.57%), while the most common neoplastic diagnosis was malignant etiology (5.71%) in radiology. Two cases showing a mismatch in the radiological and histopathological diagnoses were encountered (Table 2).

**Table 2:** Cases with a radiological and histopathological mismatch in the present study

Case	Radiology	Histopathology
1.	Hydrocele	Adenomatoid tumor
2.	Adenomatoid tumor	Angiofibroma

In the present study, 56 out of the total 70 cases were non-neoplastic lesions (80%), and 14 out of the total 70 cases were neoplastic lesions (20%) (Table 3).

**Table 3:** Distribution of study subjects according to histopathological diagnosis

Histopathological diagnosis	Frequency	Percentage
Non-neoplastic lesions		
Inflammations	04	5.68
Calcinosis cutis	01	1.42
Epididymal cyst	05	7.14
Epididymitis	01	1.42
Epididymo-orchitis	01	1.42
Fournier's gangrene with partial testicular atrophy	02	2.86
Hydrocele	06	8.57
Orchitis	02	2.86
Organized hematocele	03	4.29
Partial testis atrophy	08	11.43
Pyocele	03	4.29
Pyocele with atrophic testis	01	1.42
Pyocele with orchitis	02	2.86
Scrotal wall necrosis	01	1.42
Testicular abscess	01	1.42
Testicular atrophy	07	9.99
Undescended testis	02	2.86
Xanthogranulomatous inflammation	01	1.42
Unremarkable histology	02	2.86
Neoplastic lesions		
Adenomatoid tumor	01	1.42
Angiofibroma	01	1.42
Seminoma	07	10.00
Paratesticular liposarcoma	01	1.42
Embryonal carcinoma	02	2.86
Mixed germ cell tumor (embryonal + yolk sac tumor)	01	1.42
Groin papilloma	01	1.42
Acantholytic squamous cell carcinoma	01	1.42
Seminoma with foci of intratubular germ cell neoplasia	01	1.42
Total	70	100.0

**Figure 1:**
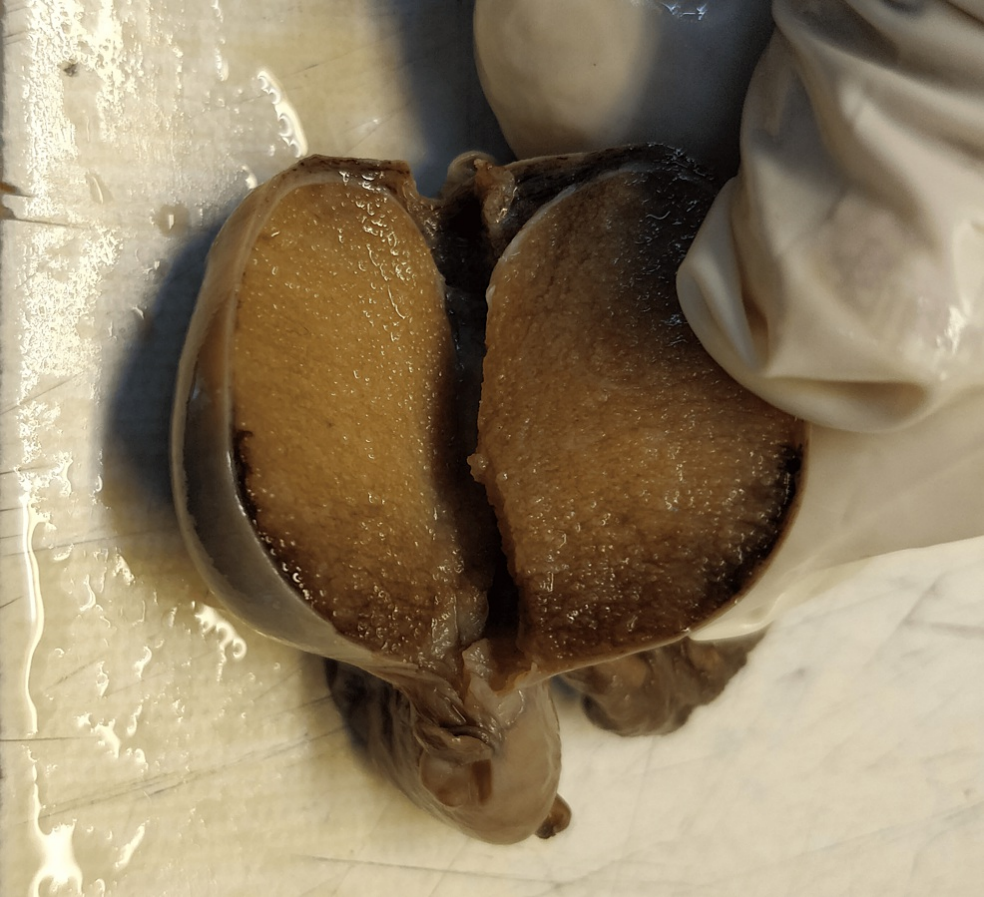
Atrophic testis Specimen of an atrophic testis with an attached cord. The testis measures 2.6 x 1.2 x 0.8cm. The patient presented with an inguinoscrotal swelling, clinically diagnosed to be undescended testis.

**Figure 2:**
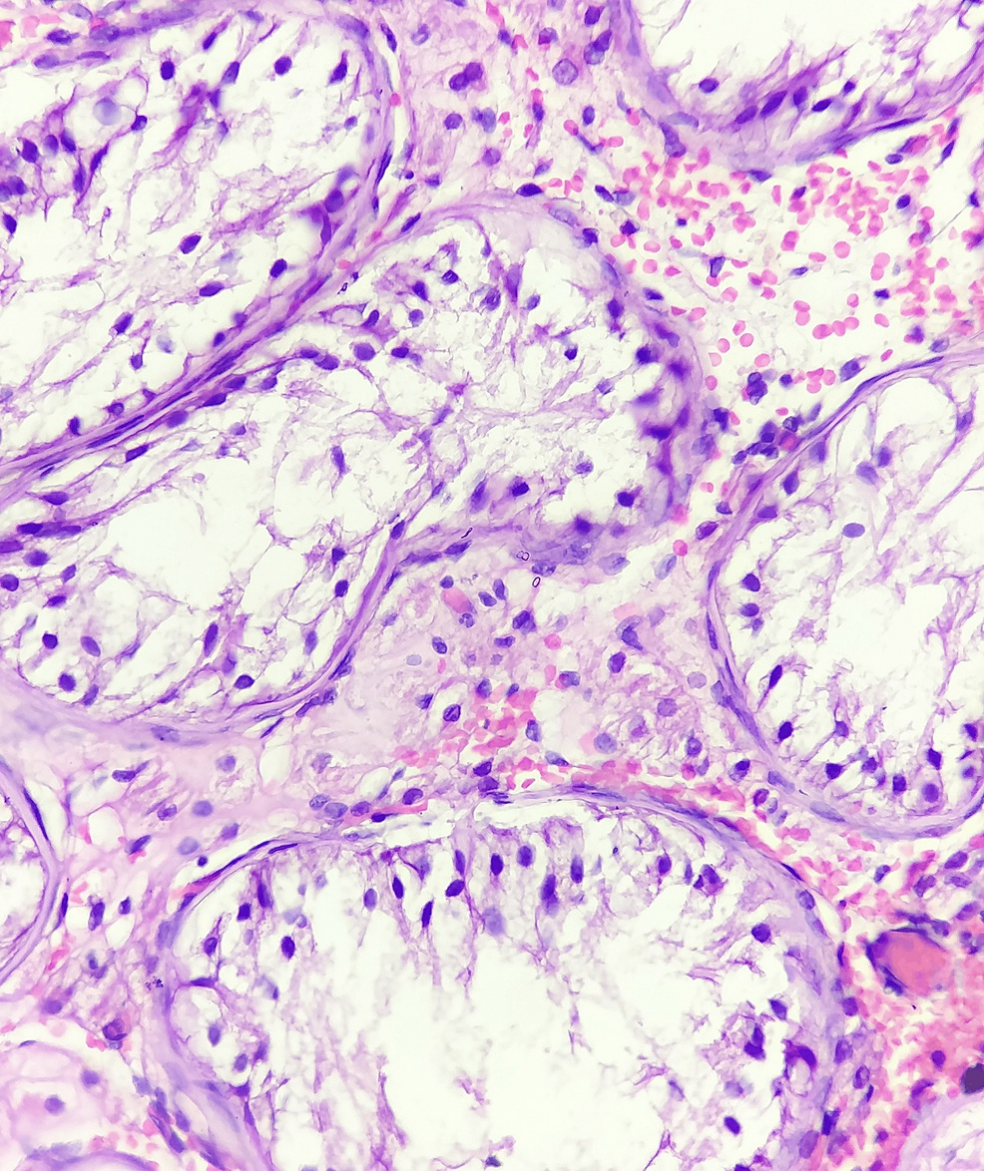
Histopathology of atrophic testis Photomicrographs of atrophic testis show seminiferous tubules lined by one to two layers of germinal cells with stromal fibrosis (hematoxylin and eosin stain, 40x).

The most common non-neoplastic lesion was testicular atrophy (partial and complete), as seen in Figures 1-2, followed by hydrocele (six cases) and epididymal cysts (five cases).

**Figure 3:**
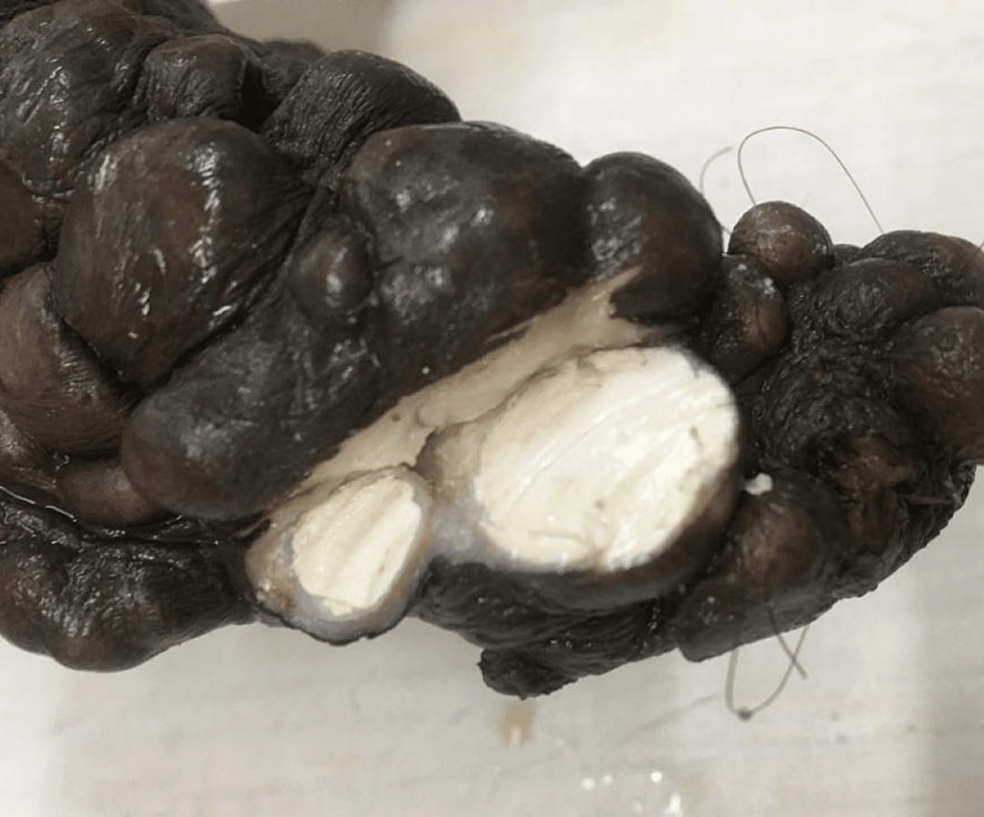
Calcinosis cutis A specimen of calcinosis cutis shows an irregular, bosselated scrotal mass. The cut surface shows multiple nodules with homogenous, whitish areas.

Hematocele, inflammations, undescended testis, testicular abscess, epididymitis, and epididymo-orchitis were some other histopathological diagnoses. One case of calcinosis cutis was observed (Figure 3).

**Figure 4:**
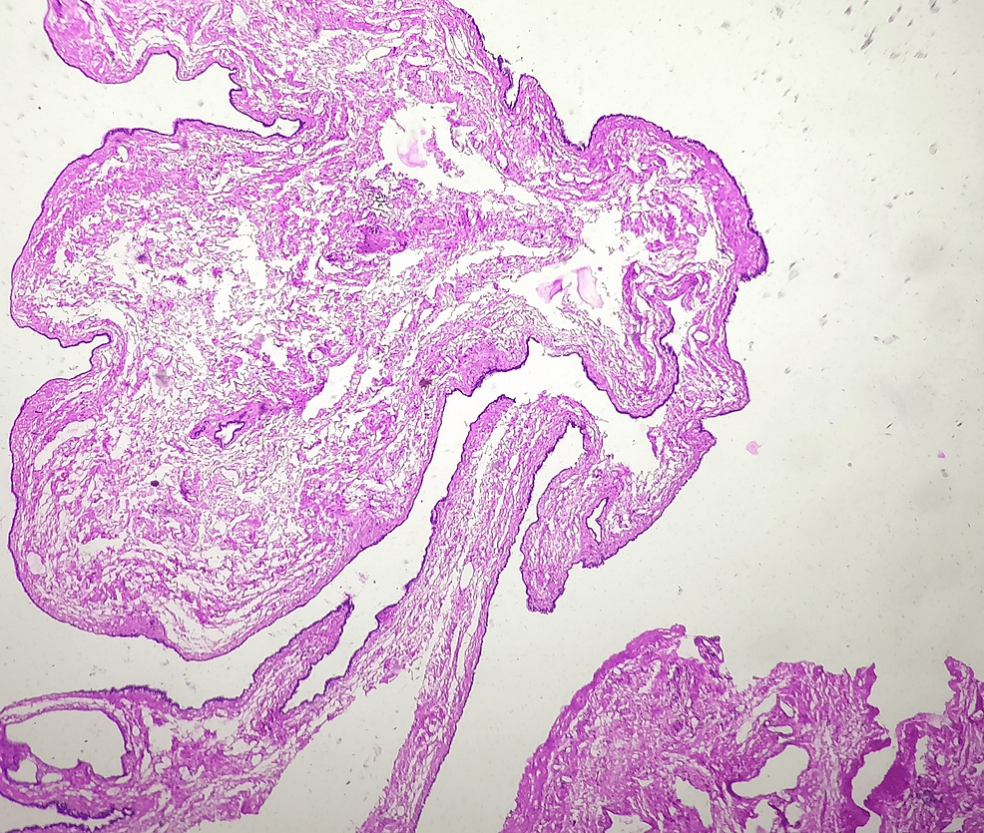
Epididymal cyst A photomicrograph reveals a fibrous cyst wall lined by flattened to cuboidal cells (hematoxylin and eosin stain, 40x).

The highest number of non-neoplastic lesions were seen in patients in the fifth decade, with hydrocele being the most prevalent diagnosis. In the fourth decade, epididymal cysts, as shown in Figure 4, are the most common diagnosis, followed by inflammation and testicular atrophy (both with two cases).

Maximum cases of testicular atrophy (partial and complete both) were found in the sixth decade, which also had maximum cases of pyocele and a single case of xanthogranulomatous orchitis.

**Figure 5:**
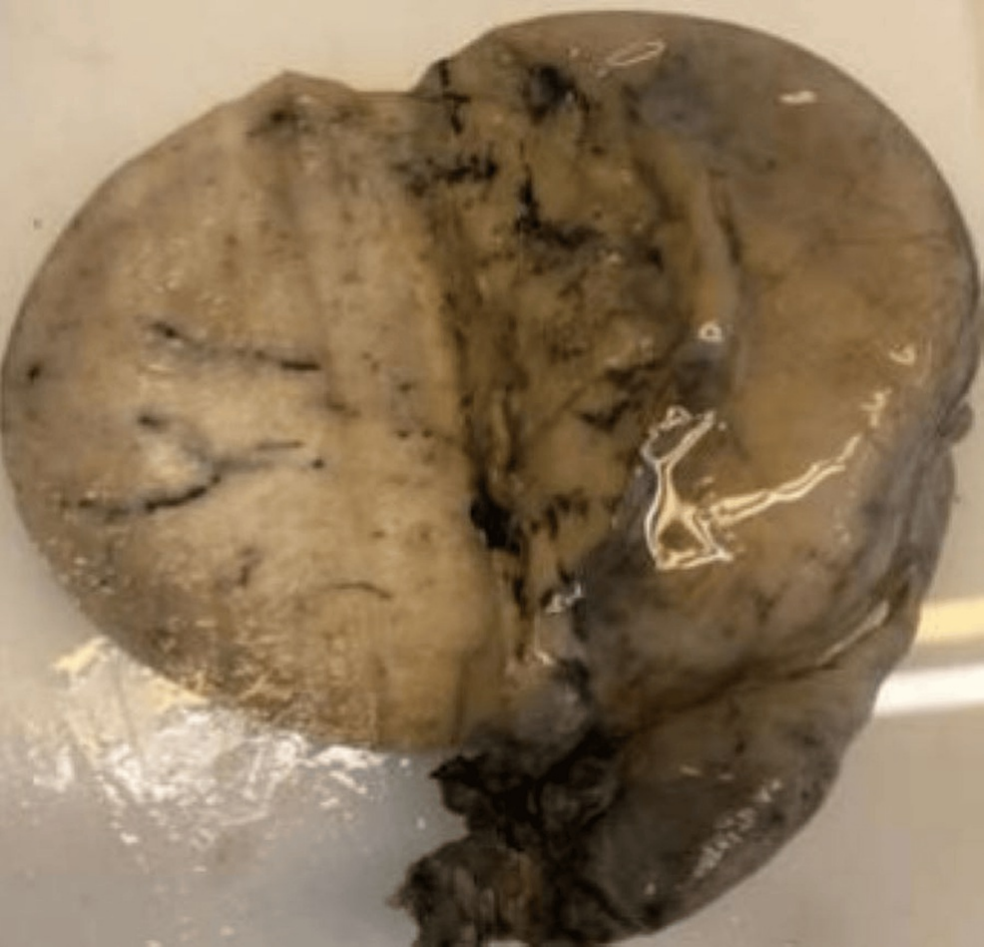
Testicular seminoma Specimen of seminoma testis showing tumor mass involving the entire testis; the cut surface is homogenous.

**Figure 6:**
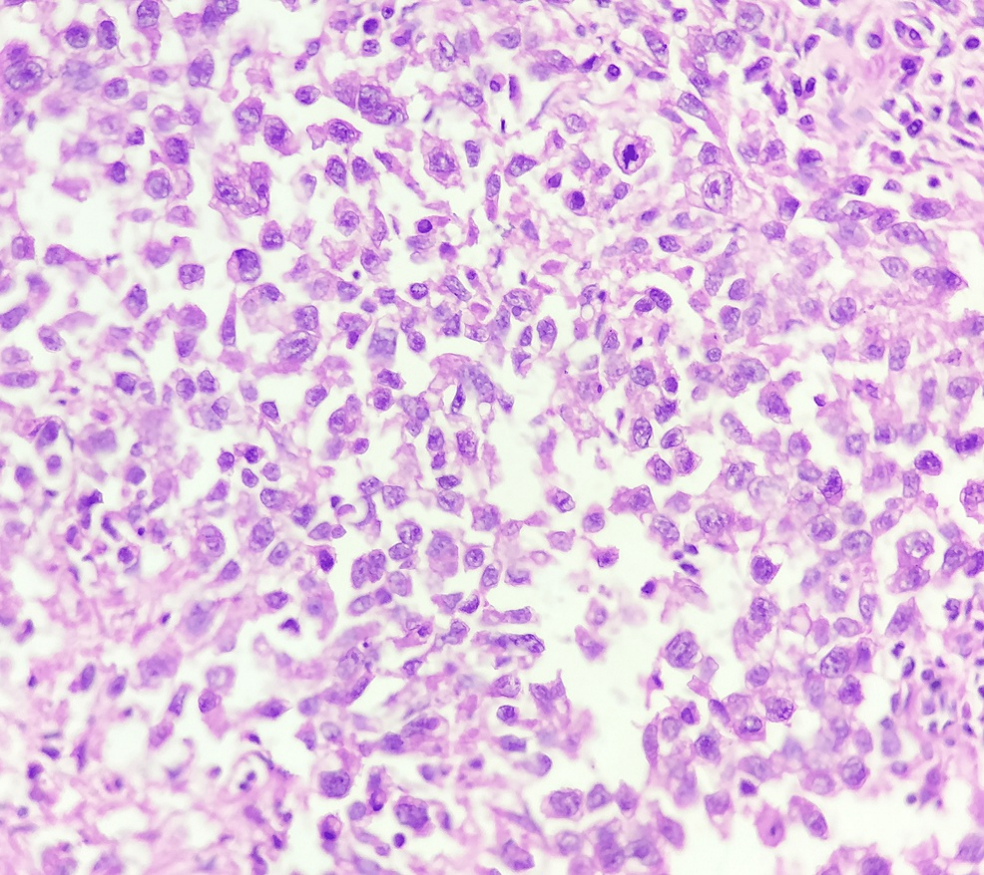
Histopathology of testicular seminoma A photomicrograph shows sheets of tumor cells separated by thin fibrous septa. The cells have clear cytoplasm with prominent nucleoli (hematoxylin and eosin stain, 40x).

The maximum number of malignant cases were with seminoma (seven cases), as seen in Figures 5-6, followed by embryonal carcinoma (two cases).

One case each of an adenomatoid tumor, angiofibroma, para-testicular liposarcoma, mixed germ cell tumor, and groin papilloma was seen. One case of seminoma was diagnosed with foci of intratubular germ cell neoplasia on histopathology. Maximum cases (six cases) were observed in the age group of 31-40 years, with seminoma (three cases) having the highest frequency. One case of seminoma was seen above 70 years of age. Four cases were seen in the fifth decade, with seminoma being the most common diagnosis, followed by embryonal carcinoma, groin papilloma, and paratesticular liposarcoma.

## Discussion

Both non-neoplastic and neoplastic diseases can affect the testis. From childhood through adulthood, there are several testicular and paratesticular lesions. The age range of 51-60 years had the most cases (23.8%). Unlike the present study, the most common age group, according to Dhawale et al. [6], Sharma et al. [3], and Baidya et al. (2017) [7], was found to be 11-20 years (second decade). Most of the non-neoplastic lesions in this study contributed to the highest number of cases in the sixth decade. This is most likely owing to the lesions' slow development and late manifestation. The youngest patient in the current study was four years old, and the oldest was 88.

In the present study, the most frequent clinical complaint was scrotal/ inguinoscrotal swelling (80% of total cases), followed by pain (41.43% of total cases). Fever was associated with 12.86% of the cases. The undescended testis (present in the upper part of the scrotal sac) was the presenting complaint in 8.5% of cases. Studies done by Dhawale et al. [6], Sharma et al. [3], and Nwafor et al. [8] reported the most common presentation to be scrotal swelling. Our study showed comparable results. Right-sided lesions were found to be more common than left-sided lesions. Bilateral lesions (five cases) included seminoma (one case), epididymal cyst (one case), organized fibrotic sac with partial testicular atrophy (one case), scrotal calcinosis (one case), and prophylactic removal of testes in a case of prostate adenocarcinoma (one case).

In this study, the maximum number of cases were non-neoplastic (80%) as compared to neoplastic lesions (20%). This concurs with most of the studies undertaken with regard to scrotal lesions. However, our conclusions differ from the study conducted by Robertson et al. [9] in the UK, which registered 31.5% non-neoplastic cases versus 68.5% neoplastic cases. A study conducted by Nikumbh et al. [10] on orchidectomy specimens in northern Maharashtra found that neoplastic lesions were 34 (57%) out of the total 60 cases as compared to non-neoplastic lesions, which were 24 (43%). Ignorance, late presentations of lesions, social taboos, and cultural rituals were proposed as responsible factors for the increased percentage of malignant cases in their study.

In the present study, the most frequent non-neoplastic diagnosis was testicular atrophy, including partial and total atrophy (a total of 16 cases). This was in concordance with Niveditha [11], who did a study on the histomorphological trends of testicular lesions and found that atrophic testis was the most prevalent non-neoplastic lesion (31.2%), followed by torsion testis (20.3%). However, Sharma et al. [3] identified undescended testis as the most prevalent non-neoplastic diagnosis in India. In this study, the maximum number of cases of testicular atrophy were found in the fifth decade. The atrophic testis was contributed by age in most of the cases, while two cases presented with undescended testis. The reason for increasing cases of undescended testis may be due to improper physical examination after birth, a lack of awareness, or ignorance. Undescended testis is a major contributing factor to the development of neoplasms.

Orchitis, non-specific epididymo-orchitis, and xantho-granulomatous orchitis were other non-neoplastic conditions diagnosed on histopathology. Tuberculous orchitis is a more prevalent type of infectious orchitis in India, as the testis and epididymis are major sites of extra-pulmonary tuberculosis. Hydrocele was the most frequent non-neoplastic para-testicular lesion in the present study. The majority of hydrocele cases were discovered between the ages of 41 and 50. In an Indian scenario, it is important to know the etiology of hydrocele, which can be due to an infection of the testis or the epididymis. Filariasis is a major cause of inguinoscrotal hydrocele in the Indian subcontinent. Most of the cases of hydrocele in our study were not associated with orchitis or epididymitis. A case of scrotal calcinosis cutis was found in a 40-year-old patient presenting with multiple cysts over the scrotum.

In 2022, a study on the burden of cancers in India based on the National Cancer Registry Program found that testicular cancer was rated 31 in India in terms of crude incidence rate among the top cancer sites in males [12]. In the previous 10 years, there has been a significant fluctuation in the incidence and mortality of testicular cancers globally [13]. When compared to European countries, Asian and African populations have a lower incidence but greater mortality. Seminoma was the most common neoplastic lesion in the present study (the maximum number of cases were in the age group of 31-40 years of age). The youngest patient with a seminoma was 30 years old, while the oldest was 85 years old. Similar findings were reported in the studies done by Patel et al. [14]. The findings of the present study are in concordance with most of the other studies. In a study conducted by Ozgun et al. [15], a mixed germ cell tumor (67.8%) was the most frequent neoplastic lesion, followed by seminoma (17.7%) and embryonal carcinoma (9.4%).

The two cases of embryonal carcinoma seen in this study were associated with raised alpha-fetoprotein (AFP) and serum lactate dehydrogenase (LDH) levels. Similarly, one example of a mixed germ cell tumor composed of embryonal carcinoma and yolk sac tumor components was linked to elevated AFP and beta-human chorionic gonadotropin (hCG) levels. Among the paratesticular tumors, one case each of paratesticular liposarcoma, adenomatoid tumor, and angiofibroma was reported in the current study.

Most of the neoplastic lesions in our study presented with painless scrotal or inguinoscrotal masses. Clinically, it is difficult to differentiate between tumors and tumor-like lesions of the testis and paratestis. Since most of them simulate testicular masses or tumors, orchidectomy becomes the surgery of choice. Hence, histopathology remains the gold standard for a correct diagnosis of scrotal lesions [16-19].

## Conclusions

The histopathological spectrum of scrotal lesions was found to be quite diverse. The majority of testicular lesions are non-tumorous, whereas tumors are uncommon, with the majority being germ cell tumors. Clinically, non-neoplastic lesions resemble neoplastic ones, with testicular swelling being the most prevalent symptom. Despite modern imaging methods and tumor marker assays, histological examination is still used to diagnose testicular and paratesticular tumors. Our study strongly recommends routine histopathological examination of all scrotal specimens (orchiectomy, excisional biopsies, and surgical removal of tumors) for the detection of various testicular and paratesticular lesions and also neoplasms, which helps in their treatment and prognosis. Thus, early management in cases of testicular and paratesticular abnormalities results in a higher life expectancy for patients with lower morbidity and death.
